# Treatment of Placenta Previa1Read before the Obstetric Section of the St. Louis Medical Society, May 25, 1909.

**Published:** 1909-09

**Authors:** Henry Schwarz

**Affiliations:** St. Louis; Professor of Obstetrics and Gynecology, Washington University


### Treatment of Placenta Previa.[1]

By HENRY SCHWARZ, M. D., St. Louis.
Professor of Obstetrics and Gynecology, Washington University.

[*St. Louis Medical Review*, July, 1909.]

IN February of the present year I read before the Alumni Association of the Washington University Medical School a paper on The Mechanism and Treatment of Placenta Previa.

On that occasion I denounced as foolish, if not criminal, all suggestions of abdominal or vaginal cesarean section as a means of treating any form of placenta previa. I likewise denounced version after Braxton-Hicks, because it deliberately sacrifices the child, and because it increases the chances for infection and because it interferes with the natural detachment of the placenta. I spoke against all attempts of forcing dilatation with steel-dilators or other means, and advised also against the intrauterine balloon-treatment, because it also requires a perforation of the placenta, thereby interfering with the natural detachment of that organ and increasing the chances for infection.

I pointed out that the majority of cases of placenta previa fall into the hands of the general practitioner, and that it is the plain duty of teachers of obstetrics to teach and to practise methods which can be employed by the general practitioner in the home of the patient.

I pointed out, that in many instances dilatation is complete, or nearly so, when the physician reaches the patient; that under such circumstances the classical version, followed by immediate extraction of the child is, as a rule, the quickest and safest mode of delivery, and that therefore students should receive careful training in the classical podalic version.

I pointed out, that in those cases in which there is insufficient dilatation or perhaps no dilatation at all, the cervical and vaginal

1. Read before the Obstetric Section of the St. Louis Medical Society, May 25, 1909.

tampon of sterile cotton, or preferably of sterile gauze, will with certainty and with safety control hemorrhage and provoke uterine contractions until dilatation is complete, when the case may be delivered by version and extraction.

I pointed out, that the tampon method is the only one which does not destroy the entirety of the ovum and which does not interfere with the natural mechanism of the first stage of labor. I insisted that students must be taught the method of packing cervix and vagina efficiently and aseptically. I illustrated a number of cases extending over the last thirty years and I stated, that out of over fifty cases of all varieties, only one mother had been lost and that her loss was due to rupture of the uterus, in a case in which a practitioner had administered a large dose of ergot after packing.

I was induced to thus emphasize my views regarding the treatment of placenta previa by the apprehension, that the unfortunate teachings of some of the leading German obstetricians, particular those of Kroenig of Freiburg, Sellheim of Tuebingen and Henkel of Greifswald, might be well received in this country and call forth another deplorable epidemic of contraindicated cesarean section.

Kroenig advocates and practises abdominal cesarean section in all cases of central placenta previa that reach the hospital before there is complete dilatation, and that have not been tamponed or otherwise temporised with by the practitioner. Sellheim recommends for the same cases the so-called extraperitoneal cervical cesarean section, and Henkel advises vaginal cesarean section for the majority of cases.

Permit me now to report in detail two cases of central placenta previa, which have come under my observation during the last few weeks, and which both furnished the ideal indication for cesarean section according to Kroenig, Sellheim and Henkel, both cases, however, did reasonably well under less heroic treatment.

CASE I.—At noon April 4, 1909, Mrs. D. entered the Washington University Hospital with the following history: The patient is 22 years old; she is married ten months; she is pregnant for the first time; her last menstruation started on August 6th, 1908. On March 23, 1909, when patient was in the thirty-third week of pregnancy, a severe hemorrhage occurred; Dr. E. H. Eyermann was called in; the hemorrhage soon subsided into moderate oozing of blood; patient was kept quietly in bed and put on bland diet and acidulated drinks; there were no uterine contractions until March 28th, when slight labor pains set in and the bleeding increased somewhat, but was not alarming. April 4, patient was transferred to the hospital; the fetal head was felt movable over the inlet; the fetal heart-sound was audible in the right lower quadrant. April 5th, at 9 p. m., there was considerable hemorrhage; the cervical canal was not unfolded and barely permitted the passage of one finger; nothing but placental tissue could be felt through the internal os. Cervical and vaginal tampon of sterile gauze; the tampon is changed five times between April 5th and April 7th. In the night of April 6th to 7th, strong labor pains set in and at 6.30 p. m., April 7th, the os is found almost fully dilated and filled out entirely by placental tissue; the patient is anesthetised; the right hand is introduced into the vagina; the placenta is peeled off from the left side of the uterine wall, until the membranes are reached; these are ruptured; the left foot is seized; the child is turned and extracted; the placenta is easily removed by Crede's method. The child, a girl, weighs 5½ pounds, is about six weeks premature and born alive. The mother is able to nurse the baby; the puerperium was uneventful; mother and child left the hospital on the 18th day and continue to do well. The placenta in this case was very large and thin; although the os was fully dilated and entirely filled out with placenta, which had to be peeled off from the uterine wall, measured at least 15 centimeters.

CASE 2.—Mrs. E. entered the hospital at 9 p.m., May 3, 1909. She is 27 years old and this is her third pregnancy; her last menstruation began September 26th, and she is therefore in the beginning of the thirty-second week of gestation. Pregnancy was uneventful until March 3, when patient bled some during the night and the following day; she remained in bed four days and was after that again undisturbed until May 3, when at 2 p. m., severe hemorrhage set in and Dr. E. H. Eyermann was called and ordered her transfer to the hospital, where she entered at 9 p. m. There was no bleeding at the time; the cervix was high up and in the left side; the degree of dilatation was not ascertained as the obstetrician on duty feared to provoke hemorrhage by forcing the finger into the cervix.

During the night there were slight uterine contractions and a scant discharge of blood; at 10 a. m., there was a free hemorrhage; a hot douche was given and cervical and vaginal gauze-tampon inserted. At 9 p. m., examination shows the fundus uteri midway between navel and xiphoid process; the fetal head rests on the left iliac fossa; the fetal heart-sounds are heard in the left lower quadrant; the packing is removed; the cervix is found high up in the left side; hard to reach; the internal os is open for one finger and filled out with placental tissue. The cervix is now packed with iodoform gauze and the vagina with plain sterile gauze. During the night there were severe uterine contractions; at 9 a. m., May 5, the packing is driven out and there is some hemorrhage; the physician on duty repacks, but not very tight, and the pains subside; at 12 noon this pack is removed; the cervix is still high up and hard to reach; shows about three centimeters of dilatation and is entirely filled out with placental tissue.

Cervix and vagina are repacked tightly and severe labor pains soon set in; when the pack is removed at 2 p. m., the os is found completely dilated (about 15 centimeters in diameter); the fetal heart sounds are no longer audible; the patient is chloroformed; the left hand is introduced into the vagina; the placenta is peeled off from the right side of the uterus; the membranes are ruptured; the right foot is seized; the child is turned and extracted; the placenta follows easy after Crede's method.

The child, a boy, was stillborn; death seemed due to the fact that the detachment of the placenta was almost complete; the placenta was of average dimensions, so that when the os was fully dilated, a margin of not more than an inch remained attached to the uterine wall. The fact that the child had barely thirty-one weeks of development accounts for its low resisting power.

Since my first protest against cesarean section as a means of treating placenta previa, a good many voices have been raised in support of safer and more conservative methods.

Professor Martin, of Berlin, attacked Kroenig and Sellheim in an open letter (*Monatsschrift fuer Geburtshilfe und Gyn.*, Bd. 28 Heft 6); Hannes, of Breslau, claims that the intrauterine balloon-treatment, as practised in Kuestner's clinics, makes all other modes of treatment unnecessary. He insists that the balloon must be placed inside of the ovum and not below it, if the best results are to be obtained (*Centralblatt fuer Gyneckologie*, 1909, No. 3).

Zimmerman, a pupil of Martin, shows that it is perfectly safe to place the metreurynter below the ovum and he has obtained excellent results. (*Centralblatt fuer Gyn.*, 1909, N. 10.)

In the United States, the most pleasing features in this question, are the conservative views expressed at New York during the meeting of the American Gynecological Society, April 20 to 22, this year. Some of these opinions state the case so plainly that I may be excused for quoting rather extensively.

Dr. Jewett, of Brooklyn, said: Even in complete previal implantation and with undilated cervix, bleeding is amenable to one or more of the usual obstetric procedures, gauze-tamponade or waterbag, within the cervix or the latter passed through the placenta, and podalic version.

Grave hemorrhage in placenta previa is due more to failure in the timely and well directed use of the obstetric measure, than to any lack of them. No shock attaches to the introduction of a hydrostatic bag and little or none to a Braxton-Hicks version.

Dr. Fry, of Washington, thinks that purely obstetric management will best meet the indication of 95 per cent. of all cases of placenta previa, but that in 5 per cent. of the cases primiparity, small vagina, rigid and undilatable cervix and placenta previa centralis will form the indication for cesarean section.

Newell, of Boston, hit the nail on the head when he said:

The advocates of cesarean section have not recognised that their personal limitations furnish the great indication for an abdominal delivery and not the exigencies of the case.

Herbert Spencer, of London, stated that he uses the Braxton-Hicks method, while his colleague uses the Champetier de Ribes bag, reducing fetal mortality considerably, with some increase in maternal mortality.

Hofmeier, of Wuerzburg, thought that abdominal section should be limited to a small percentage of cases. The old method of combined version after Braxton-Hicks, generally accepted in Germany, and the treatment by means of metreurynter in cases of large and strong children gives good results.

Pleas for cesarean section have been made recently by Allen, Baltimore, and McPherson, New York. The former in an article entitled "A Plea for the more frequent performance of cesarean section" (*American Journal of Obstetrics*, Vol. 59, No. 2), quotes Condon and says: Condon reports two operations for placenta previa with recovery of mother and infants. Condon says: There is no doubt in my mind, that in case of placenta previa centralis, when the operation of cesarean section is done early, and before temporising measures of various kinds are resorted to, the percentage of mortality can be cut down to as low a figure as ordinarily attends a simple laparotomy.

Allen also quotes Briggs, who has performed cesarean section twice for placenta previa centralis, with the result that both mothers and one infant were saved.

McPherson, in the *Bulletin* of the Lying-in-Hospital, in the City of New York (Vol. 5, No. 3), says: Regarding the utility of the operation in placenta previa, it is my belief that in cases of placenta previa with central implantation, in the presence of a firm, undilated os, where the soft parts are firm and unyielding, less damage will be done to the mother, and we shall be more certain of securing a living child by a timely cesarean section than by resorting to the other usual modes of delivery.

Of all leading textbooks the version after Braxton-Hicks is most commonly recommended.

Edgar's advice is dangerous; for rapid dilatation he recommends the Bozzi dilator. He says, that Champetier de Ribes bag or its modifications cause separation of the placenta and concealed hemorrhage and have no place in the treatment of placenta previa; to stop the hemorrhage he advises version after Braxton-Hicks.

Williams gives the following routine advice: Champetier de Ribes bag after perforation of placenta if the child is viable; if not viable, version after Braxton-Hicks; the latter is also better for private practice, because the practitioner cannot be expected to have rubber-bags. Williams's advice for exceptional cases is much better as we will see later on.

In Germany, Bumm advocates Braxton-Hicks version, although he states that intrauterine balloon treatment gives better result for the children; he thinks the fact that it requires a complicated apparatus, makes its adoption in general practice unlikely.

Kerr, of Glasgow, says: "When the cervix is dilated or dilatable to the extent of permitting two fingers being passed through it, I feel convinced that the general practitioner will be well advised to continue the treatment which has been most favored during the last thirty years, namely, version according to Braxton-Hicks.

The only textbooks in which I have found advice against the routine practice of turning according to Braxton-Hicks and which point to the good results to be obtained by the cervical and vaginal tampon, are those of Kehrer of Heidelberg, of the year 1891, and of Fritsch of Bonn, of 1904.

The highest compliment which can be paid to the efficiency of the cervical and vaginal tampon is, however, found in the books of Kerr and of Williams, although neither of them recommend it as the most rational treatment.

Kerr says: in theory I am in entire agreement with those who are opposed to the tampon, but for ordinary domestic practice, I believe it is the best treatment when bleeding has to be arrested before labor has begun and when the cervix and vagina must be plugged.

Williams says: In very exceptional cases the cervix may be so rigid, that it may be impossible to dilate it sufficiently to permit of Champetier or Braxton-Hicks. Under such circumstances, a tight cervical and vaginal pack of sterilised gause should be employed; this will check the hemorrhage for the time being and after remaining in place for a few hours will usually bring about sufficient dilatation to permit of the employment of whatever maneuvers may be deemed necessary.

The best compliment Williams pays the tampon when he speaks of the various measures for inducing labor. He says: In placenta previa, more particularly when the cervix is but slightly dilated, the use of a sterile tampon may be attended by most excellent results. In such cases, under the most rigid aseptic precautions, the end of a sterilised four-inch roller gauze-bandage is tightly packed into the cervical canal by means of uterine dressing forceps, after which the vagina is firmly and tightly packed with the same material. The pack should not be allowed to remain in place for more than twelve, or at most twenty-four hours, and on its removal at the expiration of that period.

the cervix will be found completely dilated, or at least sufficiently so to permit of other maneuvers.

It is strange that Williams knowing the value of the gauze tampon, should continue to recommend the version after Braxton-Hicks.

To summarise my convictions in regard to the treatment of placenta previa, they are as follows:

1. Neither abdominal nor vaginal cesarean section are ever indicated by any form of placenta previa.

2. Version after Braxton-Hicks, although so universally recommended, should never be used in the case of a living, viable child. When the child is not viable, this method may be used, but it is inferior to the use of the cervical and vaginal tampon or to intrauterine balloon-treatment.

3. The use of the Champetier de Ribes bag or its modifications gives good results; in the presence of a living viable child, the bag should be inserted below the placenta so as to preserve the entirety of the ovum.

4. Cervical and vaginal gauze tamponade constitutes the best method for checking hemorrhage and for provoking labor pains; it enables us to preserve the entirety of the ovum until complete dilatation is obtained, when the child should be delivered by classical version.

According to my own experience and the testimony of Williams, it is successful even in those cases which, according to Fry, form the 5 per cent. of cases indicating cesarean section and in which version after Braxton-Hicks or intrauterine balloon treatment are impossible.

It is applicable in all cases, including those which the advocates of cesarean section would reject; it requires no complicated apparatus and can be employed by every practitioner in the homes of his patients.

The cervical and vaginal gauze tamponade employed until sufficient dilatation permits of classical version and immediate delivery, is the only rational method in the presence of a living and viable child.

After the reading of this paper I became acquainted with the work of Dr. H. A. Miller, of Pittsburg, (*Amer. Jour. Surg.*, Jan., 1909).

Miller has ligated the uterine arteries in eleven cases of central placenta previa; he lost the second and the third case by delivering without waiting for the shock to pass off.

In a friendly discussion, which I had with Dr. Miller and Dr. F. F. Simpson, likewise of Pittsburg, both gentlemen were so emphatic in their statements, that after ligating both uterine arteries, dilatation will be accomplished without any further bleeding, even when it is left to nature, that I cannot but feel the ligation of the uterine arteries in certain cases will prove a valuable reserve-measure.

I do not admit Mr. Miller's claim that it ought to be practised in every case of central placenta previa, because my experience and that of others, shows, that when the case is in surroundings in which the gauze tamponade can be practised to advantage, there is no need for a method which deliberately sacrifices the life of a child; nor do I feel that it is a measure which the general practitioner can be expected to employ in the home of the patient, while the gauze tampon is.

I do, however, suggest that in proper surroundings and in skilled hands, in the presence of a viable and living child, it will add to our feeling of security, if we pass silk ligatures around the uterine arteries, without tying them; a little pull on the ligatures, by hand or by attaching a weight to them will suffice to control hemorrhage, without sacrificing the child.

440 North Newstead Avenue.

